# Post-Traumatic Stress Disorder (PTSD) Symptoms Predict Delay to Hospital in Patients with Acute Coronary Syndrome

**DOI:** 10.1371/journal.pone.0027640

**Published:** 2011-11-11

**Authors:** Jonathan D. Newman, Paul Muntner, Daichi Shimbo, Karina W. Davidson, Jonathan A. Shaffer, Donald Edmondson

**Affiliations:** 1 Department of Medicine, Columbia University Medical Center, Columbia University, New York, New York, United States of America; 2 Department of Epidemiology, University of Alabama at Birmingham, Birmingham, Alabama, United States of America; Wayne State University, United States of America

## Abstract

**Background:**

Increased delay to hospital presentation with acute coronary syndrome (ACS) is associated with poor outcomes. While demographic factors associated with this delay have been well described, scarce data are available on the role of modifiable factors, such as psychosocial disorders, on pre-hospital delay. Patients with symptoms of post-traumatic stress disorder (PTSD) often avoid stressful situations and may delay presenting for care when they experience cardiac symptoms. It is unknown, however, whether PTSD symptoms negatively impact the time to presentation during an ACS.

**Methods:**

We assessed the relationship between PTSD symptoms and pre-hospital delay in 241 adults with an ACS in the ongoing Prescription Use, Lifestyle, Stress Evaluation (PULSE) study.

**Results:**

Overall, 66% of patients were male; 40% were Hispanic or Latino. The mean age was 61.9±11.6 years old. PTSD symptoms were present in 17.8% of patients. Pre-hospital delay was longer for patients with PTSD symptoms compared to those without [geometric mean: 25.8 hours (95% CI 13.8 – 44.8) vs. 10.7 hours (95% CI 8.3 – 13.8)]; *P* = 0.005. After multivariable adjustment for age, sex, ethnicity, depression, left ventricular ejection fraction and history of myocardial infarction, the mean pre-hospital delay was 173% (95% CI: 36% –450%) longer for patients with versus without PTSD symptoms.

**Conclusion:**

Among patients presenting with an ACS, PTSD symptoms were independently associated with longer pre-hospital delays. Future studies of pre-hospital delay should examine the mechanisms underlying this association.

## Introduction

The association between pre-hospital delay prior to presentation for acute coronary syndromes (ACS) and poor outcomes has been well documented [1], [2], [3]. Studies have shown associations of older age, female gender and non-white race on longer pre-hospital delay [4], [5]. However, these factors are non-modifiable and likely account for only 10–25% of pre-hospital delay [1]. Modifiable characteristics, such as psychosocial factors and their adverse related behaviors, may help explain increases in pre-hospital delay [6], [7]. Recent studies have demonstrated that psychosocial factors are under-recognized in ACS; [8] are associated with poor outcomes following myocardial infarction (MI); [6], [9], [10] and may also influence pre-hospital delay [3], [7], [11]. Post-traumatic stress disorder (PTSD) is a risk factor for ischemic heart disease [12], [13], [14], and is associated with poor outcomes after an MI [15], but its effects on pre-hospital delay have not been examined. As patients with PTSD often avoid threatening or upsetting situations [16], [17], [18], we hypothesized they might delay presenting for care when they first experience cardiac symptoms. Therefore, we examined the relationship between prior PTSD symptoms and pre-hospital delay in patients with ACS presenting for emergency care.

## Methods

This study received ethics approval by the Institutional Review Board (IRB) of Columbia University Medical Center (# IRB - AAA9286). All patients were recruited from the clinical departments at Columbia University. Written informed consent was obtained from all study patients. Completed informed consent documents were then stored in a secure location as per Columbia University IRB protocol.

### Study Population

Patients were drawn from the Prescription Use, Lifestyle, Stress Evaluation (PULSE) study, an ongoing, single site, observational, prospective study of patients with ACS. All patients presented to the emergency department (ED) of the primary study hospital (Columbia University Medical Center) or to a nearby hospital, and were transferred to Columbia for further care. Of the 495 patients recruited between February 1, 2009 and June 14, 2010, we excluded 159 (32.1%) with direct admissions for cardiac catheterization (no pre-hospital delay because they were scheduled); 53 (9.3%) with incomplete data on symptom onset (*n* = 37, 7.5%) or triage time (*n* = 16, 2.8%); all patients missing triage time were transferred from outside hospitals with incomplete medical records and, thus, triage time could not be estimated. Finally, 31 (6.3%) patients were excluded for missing data on PTSD symptoms and 11 (2.2%) were excluded for reporting greater than 6 weeks of symptoms. After these exclusions, this analysis included 241 English or Spanish speaking patients, ≥18 years of age with an ACS, defined by study cardiologists using ACC/AHA research definitions [19].

### Main Outcome Measure

Pre-hospital delay was defined as the time between ACS symptom onset and time of ED triage. Following an approach used in prior studies, trained research staff used standardized questionnaires at the time of study enrollment to assess symptom onset [20], [21]. ED triage time was abstracted from the medical record. National guidelines for ED care of patients with chest pain recommend an electrocardiogram (ECG) on presentation or within 10 minutes of triage [22]. Time of first ECG was substituted for presentation time in 15 patients (6.2%) for whom ED triage time was not documented. Among the 226 patients with documented triage ED time, 213 (94%) had a triage to ECG interval of 10 minutes or less.

### Post-traumatic Stress Disorder Symptoms

Patients were screened within 7 days of enrollment for previous traumatic experiences and for the presence of intrusive re-experiencing symptoms of PTSD by trained mental health care professionals, using screening questions from the Structured Clinical Interview for DSM-IV Axis I Disorders-Patient Edition (SCID-I/P, Version 2) [23]. Patients were considered to have symptoms of PTSD if they reported having both a traumatic event (Criterion A) and intrusive re-experiencing of that event (Criterion B) prior to their MI. In previous research, positive symptoms for both Criterion A and B correctly identified 97% of PTSD cases [24].

### Covariates

Age, gender, and ethnicity (Hispanic or Latino vs. other) were obtained from patient interview. History of prior MI and left-ventricular ejection fraction (LVEF) during admission was determined from the medical chart. As prior studies have suggested depression and social support influence pre-hospital delay [7], [11], [20], these psychosocial factors were included as covariates. Depressive symptoms were assessed using the Beck Depression Inventory (BDI) [25], [26]. Higher BDI scores represent greater depressive symptomatology, and patients with a score ≥10 were considered to have clinically significant depression [26]. Low perceived social support (LPSS) was defined using the ENRICHD Social Support Instrument (ESSI) [26].

### Statistical Analysis

Included patients were compared on baseline characteristics to those excluded for incomplete data. Next, baseline characteristics were calculated, overall, for patients with and without PTSD symptoms. These groups were then compared using chi-square tests for categorical data and two sample t-tests for continuous variables. As in prior studies [2], [27], pre-hospital delay was positively skewed and was log-transformed for analysis. We calculated the geometric mean duration of pre-hospital delay by levels of each covariate and for patients with and without PTSD symptoms. The association between PTSD symptoms and pre-hospital delay was examined in unadjusted and age, gender and ethnicity-adjusted linear regression models. Further adjustment was performed with the addition of psychosocial (depression, LPSS) and cardiovascular (LVEF, prior MI) covariates to the age, gender and ethnicity-adjusted model. Log-transformed pre-hospital delay time was used in regression models; therefore, increases in delay are presented as percentages (ratios). Finally, to clarify the relationship between trauma exposure, PTSD symptoms and pre-hospital delay, a sensitivity analysis restricted to patients reporting prior exposure to traumatic events was performed. Specifically, among patients with exposure to traumatic events, we compared pre-hospital delay for patients with and without PTSD symptoms. All analyses were performed using SPSS, version 18 (SPSS Inc., Chicago, Illinois).

## Results

**Figure 1:**
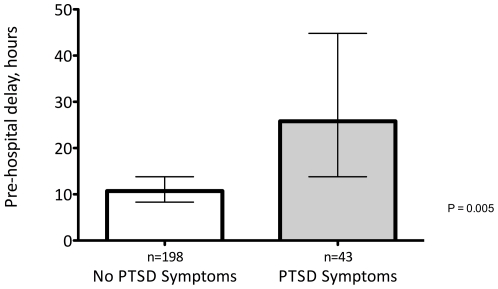
Geometric Mean Pre-hospital Delay by PTSD symptoms. *P* = 0.005 for difference in pre-hospital delay PTSD, Post-traumatic Stress Disorder.

There were no significant differences in the baseline characteristics of Table 1 between the included patients (*N* = 241) and those excluded for incomplete data (*n* = 95). Patients with PTSD symptoms (*n* = 43, 18%) versus those without (*n* = 198; 82%) were younger (mean age: 58.1 vs. 62.8 years), more likely to be depressed (70% vs. 37%), and had a higher LVEF (53.0% vs. 46.1%) (Table 1). Differences in delay time across age, gender, ethnicity, hospital presented to, prior MI, depression, LVEF, and perceived social support were not statistically significant (each p>0.10; Table 2). Pre-hospital delay was longer for patients with PTSD symptoms compared to those without (Figure 1, geometric mean: 25.8 hours [95% CI 14.9 – 44.8] vs. 10.7 hours [95% CI 8.3 – 13.8] *P* = 0.005). After age, sex, and ethnicity adjustment, patients with PTSD symptoms had a 163% (95% CI: 43% –385%) longer pre-hospital delay (Table 3). After further adjustment for depression, LVEF, prior MI and LPSS, the mean pre-hospital delay was 173% (95% CI: 36% –450%) longer for patients who had symptoms of PTSD.

**Table 1:** Characteristics of Patients by PTSD Symptoms.

Variable	Overall N = 241	PTSD Symptoms*n* = 43	No PTSD Symptoms = 198	P-value*
**Age, years**	61.9±11.6	58.1±11.7	62.7±11.4	0.02
**Male, %**	66	61	67	0.40
**Hispanic or Latino, %**	40	40	40	0.92
**Presentation to Columbia University, %**	56	51	57	0.48
**Depression** † **, %**	43	70	37	<0.01
**Myocardial Infarction, %**	63	58	64	0.31
**Prior Myocardial Infarction, %**	27	32	26	0.29
**Left Ventricular Ejection Fraction <40, %**	17	8	21	0.04
**Left Ventricular Ejection Fraction**	48.5±12.1	53.0±10.1	46.1±12.3	0.01
**Low Perceived Social Support, %**	8	7	8	0.91

Abbreviations: PTSD, post-traumatic stress disorder.

*Data are presented as percentage or mean ± S.D. comparing patients with and without PTSD symptoms.

† Depressive Symptoms defined as a Beck Depression Index (BDI) score ≥10.

**Table 2:** Geometric Mean Pre-hospital Delay by Patient Characteristics.

Variable	Delay in Hours (95% C.I.)	P-value
**Age, years**		
<65	12.2 (9.1 – 16.4)	0.53
≥65	13.1 (8.8 – 19.3)	
**Gender**		
Male	13.4 (10.0 – 17.9)	0.44
Female	11.0 (7.4 – 16.4)	
**Ethnicity**		
Hispanic or Latino	10.0 (6.9 – 14.4)	0.12
Not Hispanic or Latino	14.6 (10.7 – 19.8)	
**Hospital Presentation**		
Columbia University	12.4 (9.0 – 17.0)	0.91
Other	12.7 (9.0 – 18.0)	
**Prior MI**		
Yes	11.9 (7.5 – 18.7)	0.69
No	13.2 (10.0 – 17.5)	
**Depression** †		
Yes	11.5 (8.4 – 15.6)	0.41
No	14.0 (9.7 – 20.2)	
**LVEF ≤40%**		
Yes	14.3 (8.2 – 24.9)	0.40
No	11.0 (8.4 – 14.4)	
**Low perceived social support**		
Yes	11.7 (4.8 – 28.5)	0.84
No	12.8 (10.0 – 16.4)	

Abbreviations: MI, myocardial infarction. ACS, acute coronary syndrome

LVEF, left ventricular ejection fraction. CI, confidence interval.

† Depression defined as a BDI score ≥10.

**Table 3:** Difference in Pre-hospital Delay with vs. without PTSD Symptoms.

Model	Covariate adjustment	Percent delay (95% CI) PTSD Symptoms vs. No PTSD Symptoms	P-value
**1:**	Unadjusted	142% longer (32% to 343%)	0.005
**2:**	Age, sex, ethnicity	163% longer (43% to 385%)	0.002
**3:**	Model 2 and depression, LVEF, prior MI, LPSS	173% longer (36% to 450%)	0.005

Abbreviations: PTSD, post-traumatic stress disorder. CI, confidence interval. LVEF, left ventricular ejection fraction.

MI, myocardial infarction. LPSS, low perceived social support.

The sensitivity analysis consisted of 112 (46%) patients reporting prior traumatic events. Among these trauma-exposed patients, those with PTSD symptoms (*n* = 43), compared to those without PTSD symptoms (*n* = 69), were younger (mean age: 58.1 vs. 60.2 years), more likely to be depressed (70% vs. 35%), and had a higher LVEF (52.7% vs. 48.7%). The unadjusted pre-hospital delay time remained significantly longer for patients with PTSD symptoms when compared to trauma-exposed patients without PTSD symptoms (geometric mean: 25.8 hours [95% CI 14.9 – 44.8] vs 6.9 hours [95% CI 4.6 – 10.2] *P*<0.001). After adjustment for age, sex, ethnicity, depression, LPSS, prior MI and LVEF, the mean pre-hospital delay was 278% (95% CI: 69% –745%) longer for patients with PTSD symptoms.

## Discussion

In the current study, ACS patients with prior PTSD symptoms took more than two and a half times as long to present to the ED than did patients without PTSD symptoms. This association was present in an unadjusted model and in a model adjusted for both psychosocial and cardiovascular covariates. The reasons for the observed association between PTSD symptoms and increased delay to hospital presentation are not known. The diaphoresis, dyspnea, and tachycardia associated with PTSD may mimic ACS symptoms [28]. The self-regulatory model of behavior suggests that the cognitive and emotional representation of symptoms by PTSD patients may differ from those of non-PTSD patients, and lead to increased pre-hospital delay [29]. Also, behavioral avoidance of potentially threatening or arousing situations has been noted in patients with PTSD [16], [17], [18], and may partially explain the observed delay in presentation. It is important to note that the association between PTSD symptoms and pre-hospital delay remained after the exclusion of patients without trauma exposure from the analyses. This finding suggests that it is PTSD symptomatology – and not just the experience of traumatic events – that may increase pre-hospital delay.

The more than 10-hour longer pre-hospital delay observed, on average, in patients with prior PTSD symptoms may have clinical implications, as even a 30-minute longer delay has been associated with higher 1-year mortality rates [30]. Prior studies have suggested anxiety may be associated with an intent to delay hospital presentation [7], and it is possible that anxiety disorders, including PTSD, may represent a category of heretofore neglected modifiable factors important to pre-hospital delay.

Few studies investigating the role of psychosocial factors on pre-hospital delay have been published [6], [7]. In our study, patients with PTSD symptoms were more likely have symptoms of depression (70% versus 37% for those without PTSD). However, in contrast to prior studies [7], [11], the relationship we found between depressive symptoms and pre-hospital delay was small and not statistically significant. Prior studies that reported this association did not control for PTSD, indicating the effects of PTSD versus depression on pre-hospital delay may require further examination.

The limitations of our study include the relatively small sample size; the lack of a diagnostic PTSD assessment or a measure of symptom severity; and possible confounding by insurance status, which has been associated with pre-hospital delay [3]. However, the PTSD screening items used (history of traumatic event(s) and intrusive re-experiencing) correctly identify a high percentage of cases of PTSD [24], [31], which has been associated with adverse health outcomes. Future studies should include a diagnostic assessment for PTSD and evaluate the relationship between symptom severity and pre-hospital delay. If the association between PTSD symptoms and longer pre-hospital delay is confirmed in additional studies, trials to assess the impact of PTSD treatment on shortening pre-hospital delay may be warranted. This may have additional importance, as recent studies have suggested the prevalence of combat PTSD may be increasing [32], and have linked PTSD to atherosclerotic burden [14]. More broadly, future studies of psychosocial factors and pre-hospital delay may help guide interventions to shorten the time taken by patients to present with an ACS, as prior interventions without these factors have been largely unsuccessful [27], [33].

In conclusion, this study suggests that patients with PTSD symptoms may delay presenting for emergency care during an ACS event, and may represent a uniquely vulnerable population.
